# A Novel Liposome-Based Nanocarrier Loaded with an LPS-dsRNA Cocktail for Fish Innate Immune System Stimulation

**DOI:** 10.1371/journal.pone.0076338

**Published:** 2013-10-18

**Authors:** Angels Ruyra, Mary Cano-Sarabia, Simon A. MacKenzie, Daniel Maspoch, Nerea Roher

**Affiliations:** 1 Institut de Biotecnologia i de Biomedicina, Parc de Recerca UAB, Universitat Autònoma de Barcelona, Barcelona, Spain; 2 ICN2, Institut Catala de Nanociencia i Nanotecnologia, Campus UAB, Barcelona, Spain; 3 Institució Catalana de Recerca i Estudis Avançats (ICREA), Barcelona, Spain; “Mario Negri” Institute for Pharmacological Research, Italy

## Abstract

Development of novel systems of vaccine delivery is a growing demand of the aquaculture industry. Nano- and micro- encapsulation systems are promising tools to achieve efficient vaccines against orphan vaccine fish diseases. In this context, the use of liposomal based-nanocarriers has been poorly explored in fish; although liposomal nanocarriers have successfully been used in other species. Here, we report a new ∼125 nm-in-diameter unilamellar liposome-encapsulated immunostimulant cocktail containing crude lipopolysaccharide (LPS) from *E. coli* and polyinosinic:polycytidylic acid [poly (I:C)], a synthetic analog of dsRNA virus, aiming to be used as a non-specific vaccine nanocarrier in different fish species. This liposomal carrier showed high encapsulation efficiencies and low toxicity not only *in vitro* using three different cellular models but also *in vivo* using zebrafish embryos and larvae. We showed that such liposomal LPS-dsRNA cocktail is able to enter into contact with zebrafish hepatocytes (ZFL cell line) and trout macrophage plasma membranes, being preferentially internalized through caveolae-dependent endocytosis, although clathrin-mediated endocytosis in ZFL cells and macropinocytocis in macrophages also contribute to liposome uptake. Importantly, we also demonstrated that this liposomal LPS-dsRNA cocktail elicits a specific pro-inflammatory and anti-viral response in both zebrafish hepatocytes and trout macrophages. The design of a unique delivery system with the ability to stimulate two potent innate immunity pathways virtually present in all fish species represents a completely new approach in fish health.

## Introduction

The development of sustainable aquaculture, a strategic sector to feed the ever-increasing human population [1], relies on disease prevention through the implementation of preventive immunostimulation and effective vaccination strategies [2]. With the advent of liposomal vaccines, one can begin to conceive new non-invasive, non-stressful and easy-to-manage methods for administering immunostimulants and vaccines to a large number of cultured fish at any time of their life cycle. Liposomes are hollow spherical, safe and well-tolerated assemblies formed by a single lipid bilayer or multiple concentric bilayers that can be tailored (via selecting their composition, size, charge, etc.) to efficiently entrap a wide variety of immunostimulants and vaccines [3]. This encapsulation provides the obvious potential advantages of increasing their stability and protection, thus enhancing their immune response and disease protection, and opening up the possibility to design more efficient immunostimulant-vaccine cocktails. In addition, liposomes have been proven to act as adjuvants to potentiate immune responses alone and to be rapidly cleared from sites of administration, being preferentially distributed among macrophages [4]. Taking into account these excellent properties and since liposomes can be stable in solution or be dried [5], new opportunities will be available to aquaculture to study such systems as new immunostimulant vehicles, which could be administered either dissolved in water (immersion bath), by injection, or orally via coated-food. Herein, we describe a novel liposomal immunostimulant cocktail (hereafter called liposomal IS-cocktail) composed of two immunostimulants: the bacterial lipopolysaccharide (LPS) and the synthetic analog of dsRNA viruses, poly (I:C). Both bacterial and viral compounds were chosen to stimulate two potent innate immune pathways (TLR3 and TLR4 pathways) virtually present in all fish species [6]. The molecular basis of the immunostimulant action lies in the stimulation of innate immunity through the binding and activation of innate pathogen recognition receptors (PRRs) located on antigen-presenting cells (APCs) [7]. The principal APCs in fish are macrophages, neutrophils, dendritic cells and B cells [8], [9], [10]. Upon immunization, APCs release a variety of cytokines and chemokines regulating both innate and adaptive immunity [11]. Triggering combinations of PRRs on APCs with natural or synthetic ligands can induce synergistic activation and production of cytokines [12], [13]. Indeed, LPS is present in the cell wall of G negative bacteria and signals through TLR4 in mammals. The synthetic analog poly (I:C) (dsRNA) mimics RNA viruses and signals through TLR3 located on endosomal membranes and through RIG-I and MDA5 located in the cytosol [11]. Teleost fish can respond to dsRNA through TLR3, RIG-I and MDA5 [14] and to crude LPS preparations probably through a sensing mechanism not involving TLR4 [15]–[17], but involving peptidoglycan recognition proteins and other intracellular receptors like Nod-like receptor 3 [18]. LPS would be an excellent candidate for immunostimulation purposes, but it has been scarcely used due to its high endotoxic potential in mammals. Fish are much less sensitive to LPS toxic effects [17] and, by encapsulating LPS, we have assayed a simple way to stimulate fish innate immune system. On the other hand, the addition of dsRNA to the nanocarrier would also target anti-viral response pathways [13].

Prior to this study, some advances have been made on the encapsulation of vaccines for fish vaccination and immunostimulation. Some of these studies have suggested that microencapsulated vaccines significantly enhance the protection and immune response in various fish species [19]–[22]. Thus far, however, no one has demonstrated the ability to simultaneously control the encapsulation of several immunostimulants in unilamellar, biocompatible liposomes. Such capabilities would allow one to construct much more sophisticated and efficient liposomal immunostimulants for aquaculture. The approach used herein relies on the ability of using the surface charge of liposomes, which can be tailored by properly selecting the lipid head-groups, to optimize the encapsulation of both negatively charged LPS and dsRNA. In such design, PEGylated lipids have also been used in liposomal immunostimulant formulations to control the unilamellarity of liposomes and to prolong the plasma half-life of the immunostimulants [23], [24]. This study provides evidence that the optimized multifunctional liposomal IS-cocktail induces a concurrent anti-viral and pro-inflammatory state in zebrafish hepatocytes and trout macrophages. Moreover, insights into the mechanisms controlling the cell interaction and metabolism of the liposomes have demonstrated the possibility to target plasmatic membrane and intracellular compartments essential to achieve an optimum immune response. Our findings have also shown that the designed liposome formulations are safe at therapeutic doses and could be used in future fish health applications.

## Materials and Methods

### Materials

1,2-didodecanoyl-sn-glycero-3-phosphocholine (DLPC), 1,2-dioleoyl-sn-glycero-3-phosphoric acid monosodium salt (DOPA), Cholesterol (Chol), 3β-N-(di-methyl-amino-ethyl)carbamate hydrochloride (Cholesteryl), Cholesterol-PEG_600_ (Chol-PEG), lipopolysaccharides (LPS) from *E. coli* 0111:B4, TriReagent, insulin, EGF, chloroquine and all endocytosis inhibitors were purchased from Sigma-Aldrich. MarinaBlue-DHPE, fluorescein-DHPE, LPS-AlexaFluor594, antibiotic/antimycotic solution, TrypLE Express, Cell Mask Deep Red, Hoechst 33342 and Superscript III reverse transcriptase were purchased from Invitrogen. Poly(I:C) High Molecular Weight, poly (I:C)-Fluorescein and Primocin were purchased from InvivoGen, whereas ZFL cells were purchased from ATCC. Oligo-dT15, GelGreen and SYBR Green I were purchased from Promega, Biotium and Bio-Rad, respectively.

### Ethics statement

All experimental procedures involving rainbow trout (*Onchorynchus mykiss*) and zebrafish (*Danio rerio*) were submitted and authorized by the Ethics Committee of the Autonomous University of Barcelona (CEEH number 1582) who agree with the International Guiding Principles for Research Involving Animals (EU 2010/63).

### Preparation and characterization of liposomes of immunoliposomal formulations

Liposomal formulations were prepared by the thin film hydratation method [25] with some modifications. Briefly, DOPA, DLPC, Chol, Cholesteryl and Chol-PEG_600_ were dissolved in chloroform solutions (100 mg/ml) and mixed at the desired molar ratios (**Table 1**). The organic solvent was then evaporated by rotary evaporation to obtain a lipid film. Later, the film was hydrated with 2 ml of PBS at 0.5 mg/ml poly (I:C) or 1.5 mg/ml LPS. The encapsulation of poly (I:C) or LPS was done with an immunostimulant∶lipid ratio of 1∶30 and 1∶10, respectively. For the preparation of the liposomes that contained a cocktail of immunostimulants (hereafter called liposomal IS-cocktail), the dry lipid film was hydrated with a solution containing 0.5 mg/ml poly (I:C) and 1.0 mg/ml LPS in PBS. The co-encapsulation of poly (I:C) and LPS was done with an immunostimulant∶lipid ratio of 1∶30 and 1∶15, respectively. The resulting lipid suspensions were then vigorously shaken, and the liposomes obtained were homogenized by means of an extruder (Lipex Biomembranes, Canada) through 2 stacked polycarbonate membranes (200 nm pore size, Avanti Polar Lipids) to finally obtain unilamellar liposomes. In all cases, non-encapsulated immunostimulants were removed from liposome preparations by ultracentrifugation at 110000 *xg* for 30 min at 10°C. Liposome integrity was checked by DLS and Cryo-TEM. The particle size distribution and zeta potential (ζ) of the final liposomal formulations were measured by dynamic light scattering (DLS) using a Zetasizer Nano ZS (Malvern Instruments, UK). The morphology was examined using Cryo-Transmission electron microscopy (Cryo-TEM) in a JEOL-JEM 1400 microscope (JEOL Ltd., Japan). Liposome stability was followed (48 h at 28°C) by turbidity measurement in a Turbiscan Lab Expert (Formulaction, France).

**Table 1:** Composition and characterization of non-loaded liposomal formulations.

Name	Liposome composition		ζ′ potential (mV)	Size (nm)
NL_1,n_	DLPC 50% - Cholesteryl 35% - Cholesterol 10% - PEG5%	**++**	23.5±0.4	197.3±54.7
NL_2,n_	DLPC 50% - Cholesteryl 10% - Cholesterol 35% - PEG5%	**+**	10.4±1.8	182.7±8.4
NL_3,n_	DLPC 50% - Cholesteryl 45% - PEG5%		−5.4±1.7	204.5±21.6
NL_4,n_	DLPC 40% - DOPA 10% - Cholesterol 45% - PEG5%	**−**	−19.0±0.5	185.1±9.5
NL_5,n_	DLPC 15% - DOPA 35% - Cholesterol 45% - PEG5%	**− −**	−30.9±2.5	161.1±12.6

### Encapsulation efficiency (EE)

Encapsulation efficiencies (EE) were calculated according to the equation EE(%) = [(C_IS,total_-C_IS,out_)/C_IS,total_] x100, where C_IS,total_ is the initial immunostimulant concentration and C_IS,out_ is the concentration of non-encapsulated immunostimulant. To measure the C_IS,out_, all liposome suspensions were centrifuged at 110000 *xg* for 30 min at 10°C. Supernatant aliquots were taken to quantify the concentration of non-encapsulated poly (I:C) and LPS by UV-Vis spectroscopy using a Nanodrop ND-1000 (Thermo Scientific, USA). Poly (I:C) was linearly detected in a range from 2.5 µg/ml to 1 mg/ml (Abs at 250 nm, r^2^ = 0.999), whereas LPS was linearly detected in a range from 4.0 µg/ml to 1 mg/ml (Abs at 269 nm, r^2^ = 0.999). Liposomes that did not contain any encapsulated immunostimulant were also ultracentrifuged and their supernatant quantified (Abs at 220 nm) to verify that liposomes were properly precipitated. To calculate the EE of the liposomal IS-cocktail, the putative non-encapsulated immunostimulants in the supernatant were separated by aqueous Gel Permeation Chromatography (GPC, Ultrahydrogel 120, Waters, USA) and quantified by UV-Vis spectroscopy, where poly (I:C) and LPS were linearly detected. All experiments were done in triplicate.

### Localization of liposome-encapsulated immunostimulants

Evaluation of the distribution of encapsulated immunostimulants in liposomes was done by confocal microscopy. The liposome bilayer was labeled with MarinaBlue-DHPE (0.005 molar ratio). Fluorescent LPS-AlexaFluor594 and poly (I:C)-Fluorescein were individually or simultaneously encapsulated in liposomes and the resulting liposomal formulations were examined using a Leica TCS SP5 confocal microscope (Leica Microsystems, Germany).

### Cell culture

Zebrafish ZFL cells (CRL-2643, ATCC) were cultured at 28°C, 5% CO_2_ in Dulbecco's modified Eagle's medium (DMEM) 4.5 g/l glucose, supplemented with 0.01 mg/ml insulin, 50 ng/ml EGF, 5% (v/v) of antibiotic/antimycotic solution, 10% (v/v) heat-inactivated FBS and 0.5% (v/v) heat-inactivated trout serum (TS). HepG2 cells were grown at 37°C, 5% CO_2_ in DMEM 4.5 g/l glucose, supplemented with 5% (v/v) of antibiotic/antimycotic solution and 10% (v/v) heat-inactivated FBS. Adherent trout monocyte/macrophages were isolated as previously described [8]. Before treatments, cells were incubated 3 h in serum free medium.

### Cytotoxicity assays

Two different cell viability assays (MTT and LDH) were simultaneously performed using three cell lines (ZFL, HepG2 and primary trout macrophages). Cells were seeded at 2.5×10^5^ cells/well. The medium was removed and fresh non-supplemented medium containing the liposome formulation at indicated concentration was added, incubating the cells for 24 h. Lactate dehydrogenase (Cytotoxicity Detection Kit LDH, Roche) activity in the medium and MTT assay on the cells were performed. Cell viability was expressed as a percentage of the control. All the measurements were done in triplicate in 3 independent experiments. Dose-response curves were fitted using a sigmoidal dose-response curve model provided in the GraphPad Prism 5.0 (GraphPad software, USA). EC_50_ value was derived from these fitted curves for single experiments. Differences among data were analyzed using One-way ANOVA followed by Tukey's post test *p*<0.001.

### Endocytosis analysis using flow cytometry

To visualize liposome endocytosis, DHPE-fluorescein was incorporated at a 0.05 molar ratio into the liposomal IS-cocktail. Labeled liposomal IS-cocktail was added to either ZFL or trout macrophages to a final concentration of 750 µg/ml liposomal IS-cocktail (containing 25 µg/ml poly (I:C) and 12.5 µg/ml LPS) and incubated for selected times. After treatment, cells were cooled down, washed 3× with ice-cold PBS, trypsinized and centrifuged at 200 *xg* for 5 min. Pellets were resuspended in ice-cold PBS for FACS analysis using a BD FACSCanto cytometer (Becton Dickinson, USA). Experiments were performed in triplicate (10,000 events for each sample). The internalization of fluorescence was calculated as the mean fluorescence intensity (MFI). To compare membrane-bound versus endocyted liposomes, the medium was removed at different times (5, 15, 30 and 60 min), and the cells were washed either with ice-cold PBS (pH = 7.4) or with an ice-cold PBS-acetic acid (pH = 4.2) to remove the liposomes attached to the membrane. The remaining (internal) fluorescence of the cells was then analyzed using the PBS washed cells as a total uptake. The uptake of liposomes at long incubation times was also studied. When needed, cells were pretreated for 1 h with 100 µM chloroquine. Then, fluorescent liposomes were added and left to incubate 15 min for the ZFL cells and 30 min for the trout macrophages. After 3× PBS washes, liposome-free medium was added and cells were incubated for 1, 6 or 16 h in the presence of chloroquine, when required. Finally, cells were routinely treated for flow cytometry analysis. To determine the liposome endocytosis pathways, the following inhibitors were used: methyl-β-cyclodextrin (MβCD, 5 mM), 5-(N-Ethyl-N-isopropyl)amiloride (EIPA, 50 mM), sucrose (300 mM for ZFL, 150 mM for trout macrophages) and wortmannin (W, 100 nM). The inhibitor's toxicity was assessed (**Figure S4 in File S1**) and working concentrations were selected. Cells were pretreated for 1 h with each inhibitor, and liposomes were added for 15 min (ZFL cells) or 30 min (trout macrophages). Finally, 1 h after adding the liposomes, cells were analysed by flow cytometry.

### Endocytosis analysis using confocal microscopy

Cells were seeded one day before the endocytosis experiments. For short incubation times (from 30 min to 1.5 h), liposomal IS-cocktail was added at 750 µg/ml liposomal IS-cocktail (containing 25 µg/ml poly (I:C) and 12.5 µg/ml LPS). For the 16 h incubation time, liposomal IS-cocktail was added at 375 µg/ml liposomal IS-cocktail (containing 12.5 µg/ml poly (I:C) and 6.25 µg/ml LPS). After 3× PBS washes, cells were stained with CellMask and Hoechst and viewed under a Leica TCS SP5 confocal microscope (Leica Microsystems, Germany). Image analysis was performed using Imaris software and z-stacks were analyzed to visualize the particle contact sites and location.

### Gene expression studies

Cells were stimulated for 16 h with 750 µg/ml of liposomal IS-cocktail containing 25 µg/ml poly (I:C) and 12.5 µg/ml LPS and 375 µg/ml of liposomal IS-cocktail containing 12.5 µg/ml poly (I:C) and 6.25 µg/ml LPS. Non-loaded liposomes and non-encapsulated IS were used as controls. Total RNA from the ZFL and trout macrophages cell cultures was extracted using TriReagent following manufacturer's instructions. The RNA quality and concentration was assessed and cDNA was synthesized with 1.0 µg and 0.5 µg of total RNA for ZFL cells and macrophages, respectively, using SuperScript III reverse transcriptase and oligo-dT15 primer. PCR was carried out with 1 µl of cDNA as a template with specific primers (**Table S1 in File S1**) and qPCR was carried out using SYBR Green I mix, 500 nM of primers and 5 µl of cDNA. Samples from 3 independent experiments were run in triplicate, and quantification was done according to Livak method [26].

### TNFα secretion

Trout macrophages were incubated for 16 h with 375 µg/ml of liposomal IS-cocktail (with 12.5 µg/ml poly (I:C) and 6.25 µg/ml LPS). Non-loaded liposomes and free LPS were used as controls. Supernatants were collected, centrifuged and precipitated with 25% trichloroacetic acid. TNFα secretion was assessed by Western blot as previously described [16].

### *In vivo* toxicological assays

Adult AB zebrafish (*Danio rerio*) were held in tanks with recirculating water under a photoperiod of 14 h light/10 h dark at 28°C. Embryos were obtained from random pair-wise mating collected, rinsed and kept in E3 medium at 28°C. Viable embryos and post-hatching larvae were plated in 96-well plates. Liposomal IS-cocktail (liposome concentrations from 0.75 to 6 mg/ml) were added to the wells (200 µl), and incubated for 120 h. The plate evaporation rate was minimized as previously described [27]. Non-loaded liposomes and non-encapsulated immunostimulants were used as controls, and 24 individuals for each condition were used. Hatching rate, cumulative mortality and malformations of the embryos were recorded every 24 h, and survival curves were plotted using the Kaplan-Meier method and analysed using the log-rank test. Larvae were also frozen at −80°C and total RNA was isolated as indicated before for gene expression evaluation.

## Results

### Preparation and characterization of liposomal formulations

**Figure 1:**
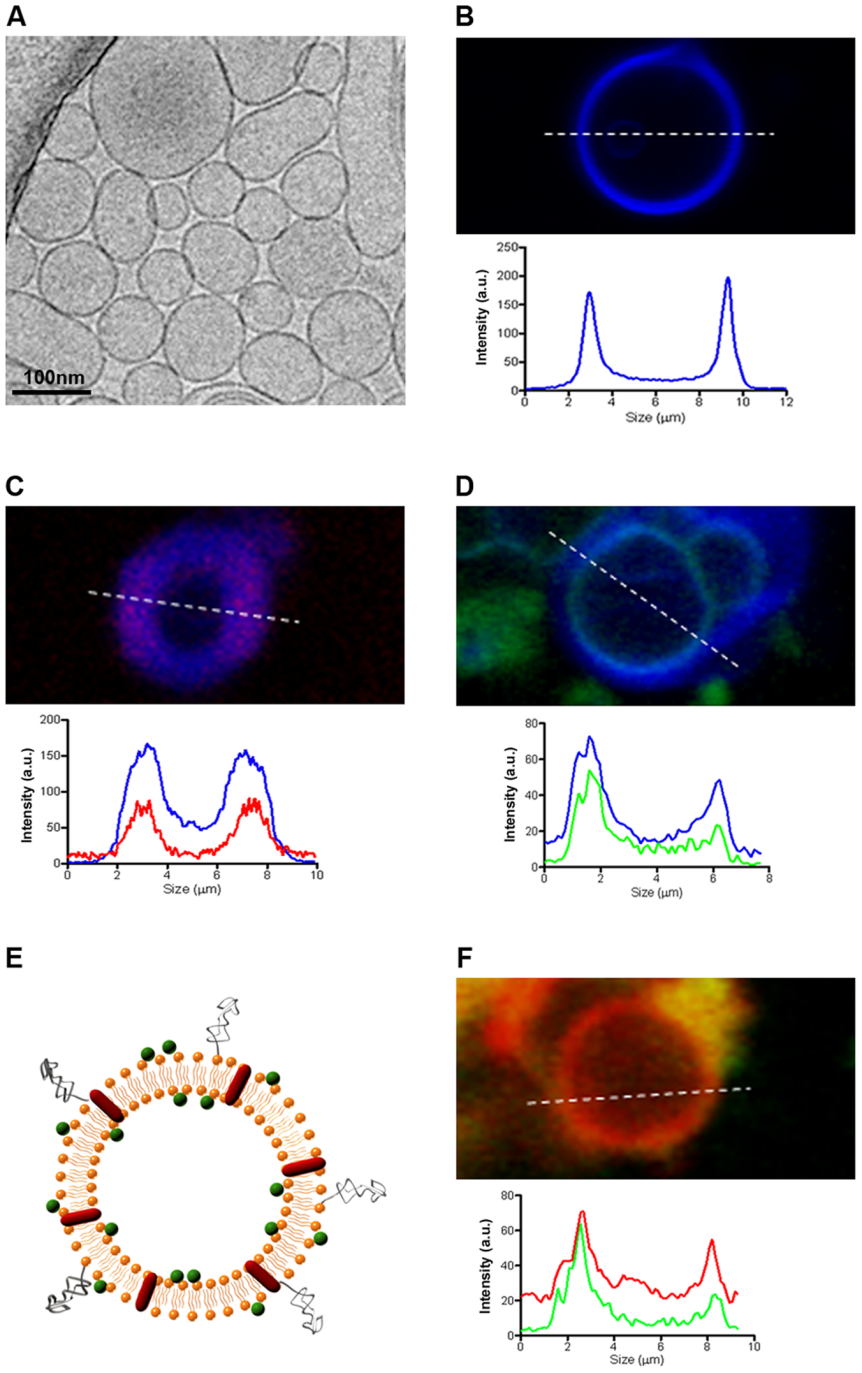
Characterization of liposomal formulations. (**A**) Representative Cryo-TEM image of DLPC/Chol/Cholesteryl/PEG_600_-Chol (5∶3.5∶1∶0.5) liposomes extruded through a 200 nm pore size membrane. (**B**) Confocal fluorescence image of a single liposome tagged on its lipid bilayer with Marina Blue-DHPE (blue) and its corresponding fluorescence intensity profile. (**C**) Confocal fluorescence image of a single Marina Blue-labeled liposome containing AlexaFluor594-labeled LPS (red) and their corresponding fluorescence intensity profiles. (**D**) Confocal fluorescence image of a single Marina Blue-labeled liposome containing fluorescein-labeled poly (I:C) and their corresponding fluorescence intensity profiles. (**E**) Schematic representation of the liposomal IS-cocktail (NL_c_) showing the presence of both encapsulated LPS (red) and poly (I:C) (green) in the lipidic bilayer of liposomes. (**F**) Confocal fluorescence image of a single liposome containing both fluorescein-labeled poly (I:C) (green) and AlexaFluor594-labeled LPS (red) and their corresponding fluorescence intensity profiles.

Series of liposomal formulations with different lipid membrane composition and net surface charges were prepared to determine the optimal liposomal formulation to achieve the maximum encapsulation efficiency of LPS and poly (I:C). Three lipid mixtures were studied, NL_1,n_ and NL_2,n_, formed by the cationic lipid mixture of DLPC- Cholesteryl-Chol-PEG, NL_3,n_, constituted by the neutral mixture DLPC-Chol, and NL_4,n_ and NL_5,n_, formed by the anionic lipid mixture DLPC-DOPA-Chol-PEG (**Table 1**). In all formulations, small unilamellar vesicles (**Figure 1A**) were obtained with a mean size ranging from 161.1±12.6 nm to 204.5±21.6 nm. In all cases, a 5% of Chol-PEG_600_ was included to achieve uniform samples. Encapsulation efficiencies of LPS or poly (I:C) in the different NL_1,n_ to NL_5,n_ formulations were studied, showing that a positively charged liposome surface, like in NL_1,n_ (+23.47±0.40 mV) and NL_2,n_ (+10.43±1.77 mV), favors the encapsulation of LPS and poly (I:C). In contrast, the encapsulation efficiency of both LPS and poly (I:C) in liposomes decreased as the surface charge became more negative like in NL_5,n_ (−30.87±2.53 mV), as previously described by Balazs *et al.* and Nakhla *et al.* [28], [29]. It has been suggested that the attractive interaction between the negative charge of the immunostimulants and the positive charge of the liposome surface results in near-perfect conditions to achieve the highest encapsulation efficiencies [30]. For example, the influence of these interactions to the encapsulation of both LPS and poly (I:C) was further confirmed by a decrease of the positive ζ′ potential value down to −4.34±0,41 and 4.5±1.1 for both NL_2,LPS_ and NL_2,poly (I:C)_, respectively. The maximum loading efficiencies for LPS were 49.6±5.9% and 66.0±0.1% for NL_2,LPS_ and NL_1,LPS_, respectively. Interestingly, loading efficiencies achieved for poly (I:C) were always higher, with values of 95.0±1.4% and 91.2±0.1% for NL_2,__poly (I:C)_ and NL_1,__poly (I:C)_, respectively (**Table 2**). To further characterize the physicochemical structure of such cationic liposomal formulations, we encapsulated AlexaFluor594-labeled LPS (**Figure 1C**) and fluorescein-labeled poly (I:C) (**Figure 1D**) into Marina Blue-labeled liposomes (**Figure 1B**). Confocal microscope images of non-extruded liposomes demonstrated that both LPS and poly (I:C) were incorporated into their lipidic bilayer. Figures 1C–D show the spatial superimposition between fluorescence intensities of AlexaFluor594-LPS and Marina Blue-liposomes (**Figure 1C**) as well as of fluorescein-poly (I:C) and Marina Blue-liposomes (**Figure 1D**), further confirming that both immunostimulants are localized in the lipidic bilayer of cationic liposomes. Next, we investigated the cytotoxicity of cationic liposomes without encapsulated immunostimulants of both, NL_1,n_ and NL_2,n_ formulations, showing the maximum loading efficiencies (**Figure S1 in File S1**). Both types of liposomes were *in vitro* assayed on ZFL and HepG2 cell lines using MTT and LDH assays. Interestingly, NL_1,n_ and NL_2,n_ liposomes showed similar cytotoxicity activity in HepG2 cells (**Figure S2 in File S1**). However, the more cationic liposomes (NL_1,n_) clearly showed higher toxicity on ZFL cells (EC_50_ = 0.166 mg/ml) than the less cationic one (NL_2,n_). Because of their similar loading efficiencies but different cytotoxicity, the less toxic NL_2,n_ formulation (DLPC 50%-Cholesteryl 10%-Chol 35%-Chol-PEG 5%) was finally chosen as the ideal liposomal composition to co-encapsulate LPS and poly (I:C) (**Figure 1E**). Using these conditions, the resulting liposomal IS-cocktail (hereafter referred to as NL_c_ formulation) was composed of 125.8±6.6 nm-in-diameter liposomes that entrapped both LPS and poly (I:C) with loading efficiencies of 22.3±2.1% and 99.6±0.1%, respectively. Therefore, the NL_c_ formulation was composed of a mixture of 15 mg/ml of liposomes containing 250 µg/ml and 500 µg/ml of LPS and poly I:C, respectively. Importantly, after co-encapsulating LPS and poly (I:C), such liposomes exhibited a slightly positive surface charge (1.37±3.58 mV), which was attributed to electrostatic interactions between their positively charged lipidic bilayer and the negatively charged immunostimulants. The occurrence of these attractive interactions was corroborated by co-encapsulating AlexaFluor594-labeled LPS and fluorescein-labeled poly (I:C) into cationic liposomes, from which the localization of both immunostimulants in the lipidic bilayer was observed (**Figure 1F**).

**Table 2:** Efficiencies for the encapsulation of LPS and poly (I:C).

Name	EE LPS (%)	EE poly (I:C) (%)
NL_1,LPS_	66.0±0.1	
NL_1, poly (I:C)_		91.2±5.9
NL_2,LPS_	49.6±5.9	
NL_2, poly (I:C)_		95.0±1.4
NL_3,LPS_	6.9±0.4	
NL_3, poly (I:C)_		25.8±7.6
NL_4,LPS_	5.9±3.2	
NL_4, poly (I:C)_		38.0±4.5
NL_5,LPS_	2.0±1.3	
NL_5, poly (I:C)_		12.9±4.3

Encapsulation efficiencies (EE) for separately encapsulating an initial concentration of 1.5 mg/ml of LPS and 0.5 mg/ml of poly (I:C) into 15 mg/ml of the liposomal (NL_1–5_) formulation.

### Evaluation of cell toxicity of liposomal NL_2,LPS_, NL_2,poly (I:C)_ and NL_c_ formulations on zebrafish hepatocytes and trout macrophages primary cultures

**Figure 2:**
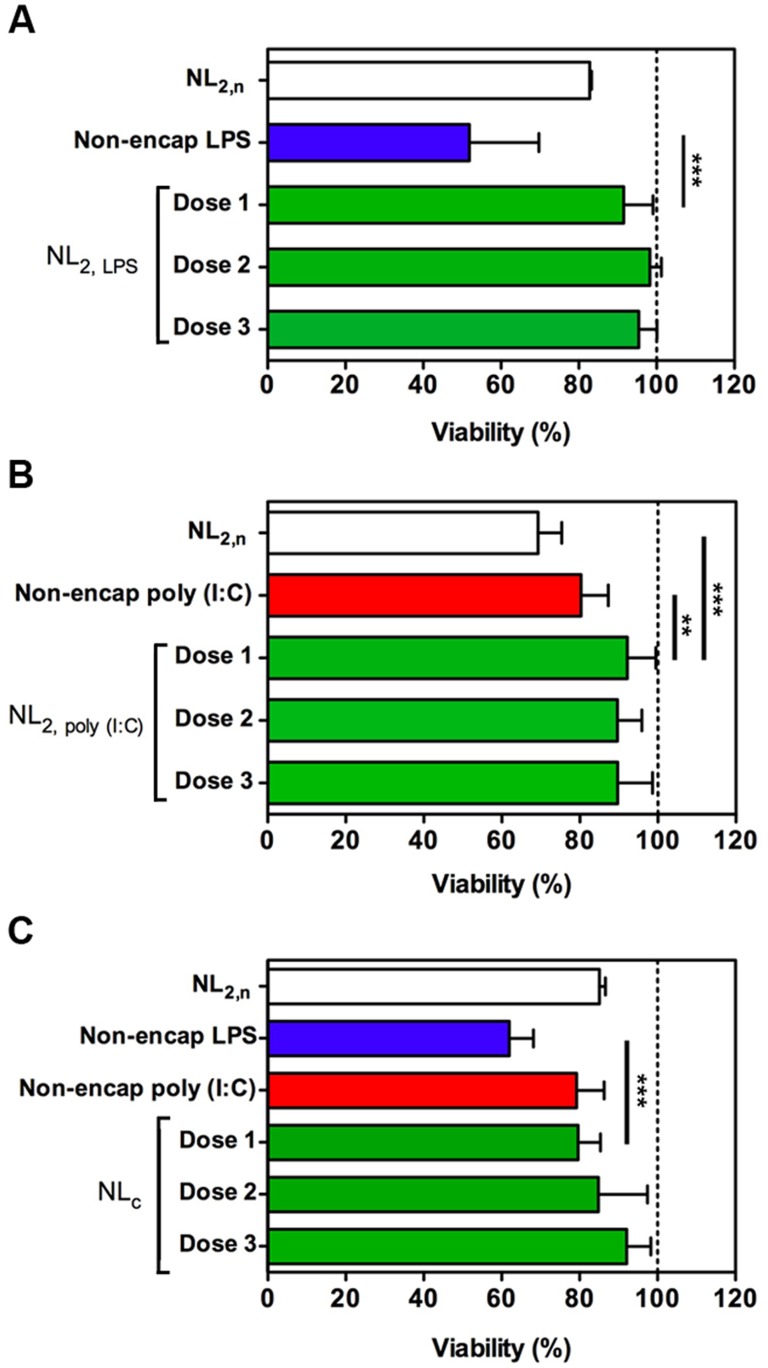
Cytotoxicity of NL_2, LPS_, NL_2, poly (I:C)_, and NL_c_ formulations in ZFL cells by MTT-based assay. (**A**) Viability of ZFL after 24 h incubation with liposome-encapsulated LPS (NL_2, LPS_, green bars) at Dose 1 = 1 mg/ml liposome with 50 µg/ml LPS, Dose 2 = 0.5 mg/ml liposome with 25 µg/ml LPS and Dose 3 = 0.20 mg/ml liposome with 10 µg/ml LPS. The white bar is the empty liposome control (NL_2,n_, 1 mg/ml liposome) and the blue bar is the free LPS control (50 µg/ml). (**B**) Viability of ZFL after 24 h incubation the liposome-encapsulated poly (I:C) (NL_2, poly (I:C)_, green bars) at Dose 1 = 1.5 mg/ml liposome with 50 µg/ml poly (I:C), Dose 2 = 0.75 mg/ml liposome with 25 µg/ml poly (I:C) and Dose 3 = 0.375 mg/ml liposome with 10 µg/ml poly (I:C). The white bar is the empty liposome control treatment (NL_2,n_, 1.5 mg/ml liposome) and the red bar is the non-encapsulated poly (I:C) control (50 µg/ml). (**C**) Viability of ZFL cells after 24 h incubation with liposomal LPS-poly (I:C) cocktail (NL_c_, green bars) at Dose 1 = 1.5 mg/ml liposome with 50 µg/ml poly (I:C) and 25 µg/ml LPS, Dose 2 = 0.75 mg/ml liposome with 25 µg/ml poly (I:C) and 12.5 µg/ml LPS and Dose 3 = 0.375 mg/ml liposome with 12.5 µg/ml poly (I:C) and 6.25 µg/ml LPS. The white bar is the empty liposome control treatment (NL_2,n_, 1.5 mg/ml liposome), the blue bar indicates the free LPS (25 µg/ml) and the red bar is the free (I:C) control (50 µg/ml). Non-treated cells were used as 100% viability control (dotted line). Data represent means ± SD of three independent experiments. Differences were analyzed using One-way ANOVA followed by Tukey's post test. **, *p*<0.01; ***, *p*<0.001.

To fully characterize the safety of our formulations, we carried out *in vitro* cytotoxic studies (**Figure 2** **and Figures S2, S3 in File S1**). The therapeutic immunostimulant doses were chosen according to our previous results on LPS and poly (I:C) responses in different fish species [16], [31]. Based on these results, dose-response experiments were conducted with NL_2,n_, NL_2,LPS_, NL_2,poly (I:C)_ and NL_c_ in ZFL cells at the indicated concentrations (**Figure 2**). None of the encapsulating formulations showed toxicity at potential therapeutic doses in these cells. Moreover, free LPS toxicity (50 µg/ml LPS, 51.8%±17.9 viability and 25 µg/ml LPS, 62.0%±6.01 viability) was avoided by nanoencapsulation. Also, poly (I:C) treatment prompted a slight decrease in viability (50 µg/ml poly I:C, 80.32%±7.01 viability) that was fully reverted when this molecule was encapsulated (**Figure 2B**). Further, empty NL_2,n_ showed low toxicity but higher than NL_c_ in all cases, which can be attributed to changes suffered by the liposomes after the encapsulation of LPS and poly (I:C) that further improve its biocompatibility. The same results were obtained by using the LDH assay (**Figure S2 in File S1**). Finally, toxicity studies were also carried out using trout primary cell cultured APCs (**Figure S3 in File S1**). In this cells, we observed low toxicity levels of NL_c_ formulations (20% over basal mortality), but did not observe a LPS/poly (I:C) mediated toxicity at the indicated doses.

### Endocytosis of NL_c_ formulation by ZFL cells and trout macrophages primary cultures

**Figure 3:**
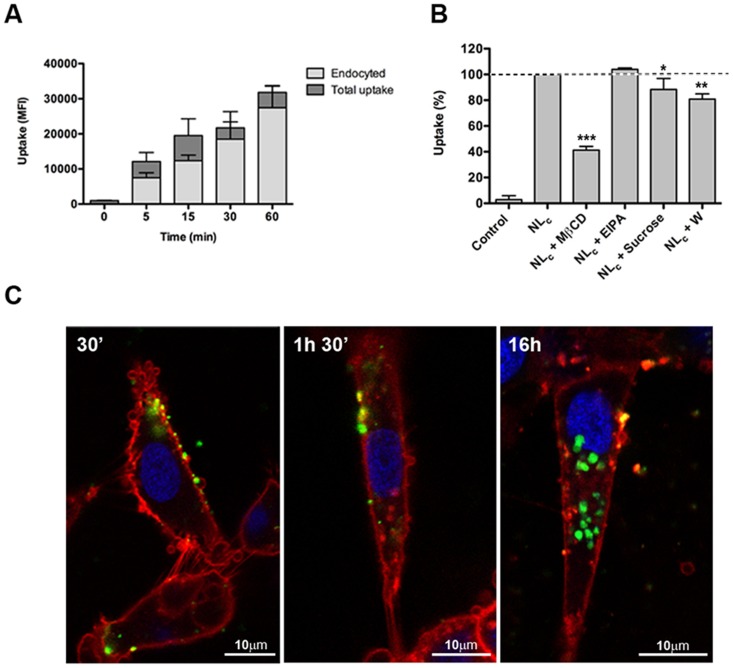
Endocytosis of NL_c_ formulation by ZFL cells. (**A**) Flow cytometry time-course comparison of the membrane-bound (dark grey bar) versus the endocyted liposomes (light grey bar) after incubation with NL_c_ (750 µg/ml liposome, 25 µg/ml poly (I:C) and 12.5 µg/ml LPS) at the indicated times. Data represent means ± SD of three independent experiments. (**B**) Effect of chemical inhibitors on the endocytosis of the NL_c_ (750 µg/ml liposome, 25 µg/ml poly (I:C) and 12.5 µg/ml LPS). Inhibitors were used at the following concentrations: MβCD at 5 mM, EIPA at 50 µM, sucrose at 300 mM and W at 100 nM. The uptake of cells without inhibitors (NL_c_ bar) was used as 100% uptake control and non-treated cells were used as control (control bar). Data represent means ± SD of three independent experiments. Differences were analyzed using One-way ANOVA followed by Tukey's post test. *, *p*<0.05; **, *p*<0.01; ***, *p*<0.001. (**C**) Confocal microscopy images of fluorescent liposomes (NL_c_) endocyted by ZFL cells. Cells were incubated for 30 min, 1.5 h and 16 h with NL_c_ containing DHPE-Fluorescein (green) at a 0.05 molar ratio. Cell membranes were stained with CellMask (red) and the nucleus was stained with Hoechst (blue).

Since hepatocytes play a major role in physiological detoxification processes and APCs are the key targets of our liposomes, we next evaluated the liposome uptake in both systems using flow cytometry and confocal microscopy. In ZFL cells, we observed a rapid (5 min) and efficient liposome uptake (**Figure 3A**) that reached a maximum in 1 h, and then started to decrease during the next 16 h, indicating that NL_c_ were probably metabolized by the endosomal/lysosomal system. Different studies have shown the ability of cationic liposomes to deliver different compounds to endosomal compartments [12], [32]. To further explore this process, we assayed the NL_c_ endocytosis in the presence of chloroquine (CQ), an inhibitor of lysosomal acidification, and we observed a significant increase of fluorescence in the presence of CQ (**Figure S5A in File S1**). This observation confirmed the occurrence of NL_c_ in the endosomal/lysosomal compartment (55.53±0.83% CQ-dependant endocytosis inhibition at 16 h). To discriminate between membrane-bounded and endocytosed NL_c_, we measured the total versus endocytosed fluorescence at different times, observing that around 80% of total fluorescence signal corresponded to endocytosed liposomes (**Figure 3A**) that accumulated intracellularly forming cytosolic agglomerates of 1.13±0.42 µm mean size (**Figure 3C**). To distinguish between the various mechanisms of endocytosis, a series of FITC-labelled NL_c_ liposome uptake assays were performed in the presence of inhibitors (methyl-β-cyclodextrin, MβCD, sucrose, wortmannin and EIPA) known to block a particular endocytosis pathway (**Figure 3B**). Treatment of cells with MβCD, a caveolae-mediated endocytosis inhibitor, led to a 60±5.9% (p<0.001) decrease in liposome uptake, whereas treatment with the macropinocytosis inhibitors wortmannin and EIPA provided contradictory results. While wortmannin inhibited uptake (19±4%; p<0.01), EIPA, a more specific macropinocytosis inhibitor, did not. The PI3K inhibitors (e.g wortmannin) have been described to have pleiotropic effects on endocytosis as they can block the internalization of ligands of the clathrin- and caveolae- mediated pathways [33], [34]. Thus, in ZFL cells, wortmannin could affect caveolae-mediated endocytosis instead of macropinocytosis. Finally, treatment with hypertonic medium (sucrose) led to a 15±6% (p<0.05) inhibition, indicating that clathrin-mediated endocytosis may also contribute to NL_c_ uptake. All these data suggested that NL_c_ could be endocytosed by ZFL cells mainly through the caveolae-dependent endocytosis pathway, but clathrin-mediated endocytosis could also be involved in liposome uptake.

**Figure 4:**
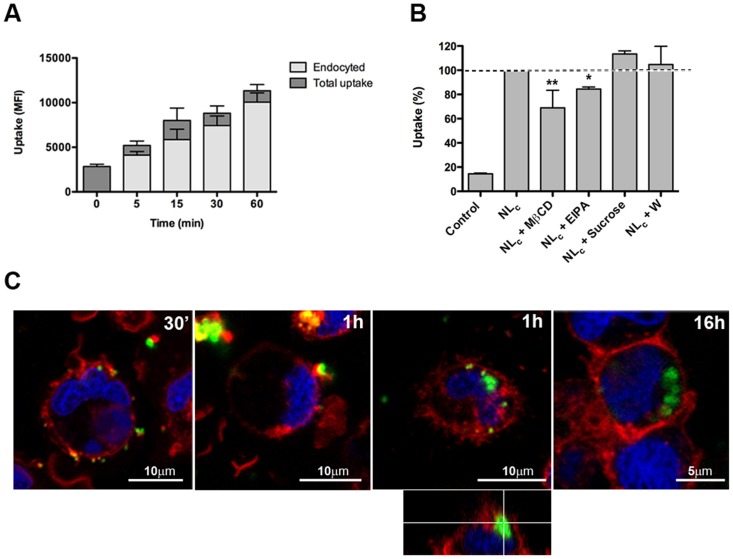
Endocytosis of NL_c_ formulation by trout macrophages. (**A**) Flow cytometry time-course comparison of the membrane-bound (dark grey bar) versus the endocyted liposomes (light grey bar) after incubation with 750 µg/ml liposome-encapsulated 25 µg/ml poly (I:C) and 12.5 µg/ml LPS at the indicated times. Data represent means ± SD of three independent experiments. (**B**) Effect of chemical inhibitors on the endocytosis of NL_c_ (750 µg/ml liposome-encapsulated 25 µg/ml poly (I:C) and 12.5 µg/ml LPS) macrophages uptake. Inhibitors were used at the following concentrations: MβCD at 5 mM, EIPA at 50 µM, sucrose at 150 mM and W at 100 nM. The uptake of cells not treated with inhibitors (NL_c_ bar) was used as 100% uptake control and non-treated cells were used as control (control bar). Data represent means ± SD of 3 independent experiments. Differences were analyzed using One-way ANOVA followed by Newman-Keuls post-test. *, *p*<0.05; **, *p*<0.01. (**C**) Confocal microscopy images of fluorescent liposomes (NL_c_) endocyted by macrophages. Cells incubated 30 min, 1 h and 16 h with NL_c_ containing DHPE-Fluorescein (green) at a 0.05 molar ratio. Cell membranes were stained with CellMask (red) and nucleus with Hoechst (blue).

The uptake in differentiated trout macrophages was also evaluated. As shown in Figure 4, these cells were able to efficiently endocyte NL_c_. We measured total versus intracellular fluorescence by flow cytometry, and similarly to ZFL cells, macrophages were able to internalize around 80% of fluorescent liposomes in 1 h (**Figure 4A**). In contrast to ZFL cells, however, macrophages did not metabolize liposomes in the endosomal/lysosomal compartment since we could detect the same fluorescence levels even 24 h later (**Figure S5 in File S1**). Note that the intracellular liposomes, as in ZFL cells, were present primarily in the cytosol as agglomerates (1.09±0.37 µm), with no fluorescence in the nuclei (**Figure 4C**). Again, we performed a series of liposome uptake assays in the presence of inhibitors, and we observed that in macrophages both MβCD and EIPA were able to inhibit the endocytosis by 31.09±14.52% (p<0.01) and 15.57±1.72% (p<0.05), respectively (**Figure 4B**). These results indicated that caveolae-mediated endocytosis and macropinocytosis/phagocytosis are the main endocytic pathways for liposome internalization in trout macrophages.

### The immunostimulatory effects of NL_c_ formulation on ZFL cells and trout macrophages

**Figure 5:**
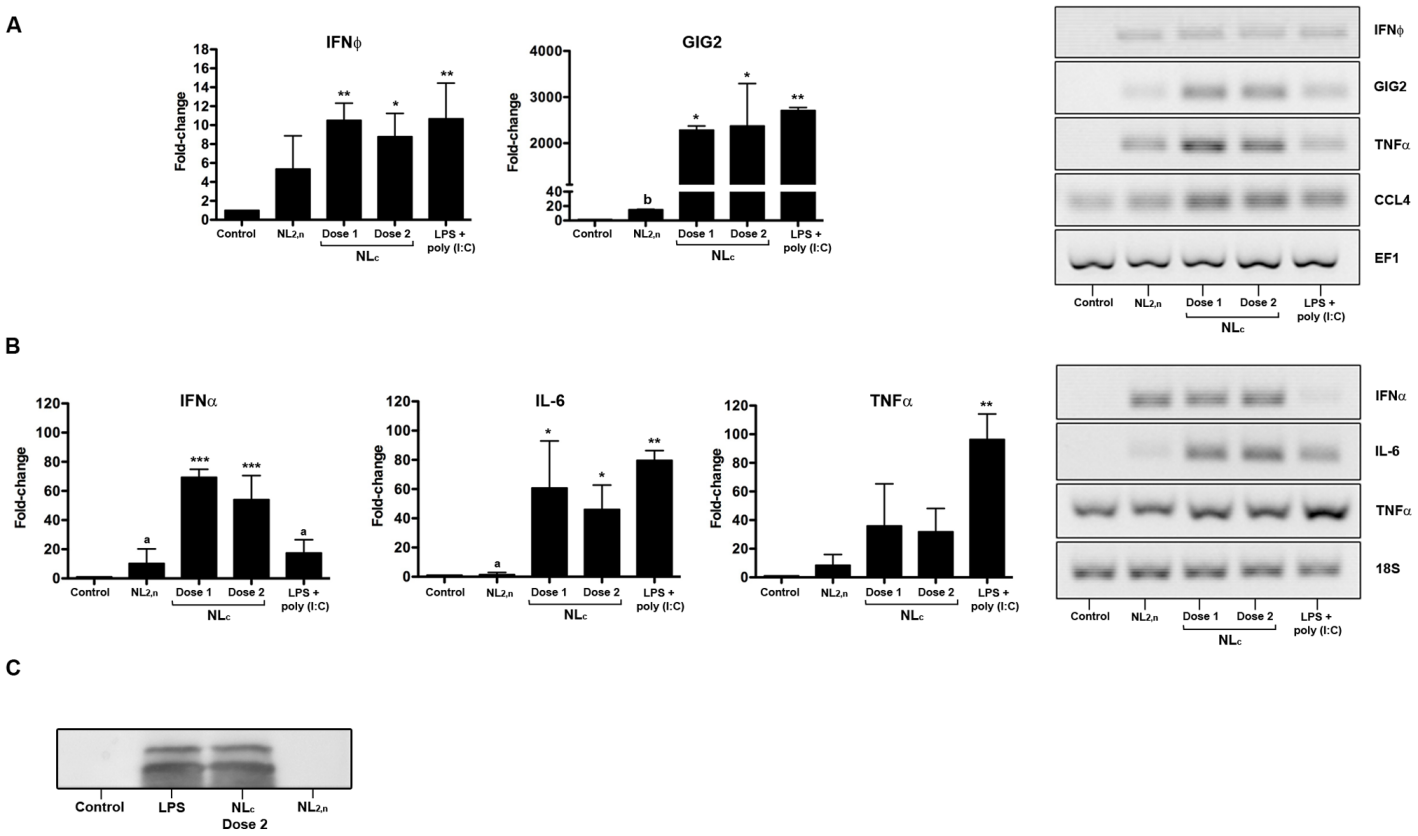
Analysis of gene expression in ZFL cell culture (A) and trout macrophage primary cell culture (B) after 16 h exposure to liposomes. NL_2,n_ = liposomes without immunostimulants (750 µg/ml), NL_c_ Dose 1 = liposomes (750 µg/ml) containing 25 µg/ml poly (I:C) and 12.5 µg/ml LPS, NL_c_ Dose 2 = liposomes (375 µg/ml) containing 12.5 µg/ml poly (I:C) and 6.25 µg/ml LPS, and LPS+poly (I:C) = stimulation control (25 µg/ml poly (I:C), 12.5 µg/ml LPS). Elongation factor (EF1) was used as reference gene for ZFL cells and 18S for trout macrophages. IFN (φ for ZFL and α for macrophages), GIG2, CCL4, IL-6 and TNFα abundance was analyzed by Q-PCR (left panel) and conventional PCR (right panel). Data represent means ± SD of 3 independent experiments. Values with asterisk are statistically significant relative to the control (*, *p*<0.05; **, *p*<0.01; ***, *p*<0.001) and values with letters (_a_,_b_) are statistically significant relative to NL_c_ Dose 1 (_a_, *p*<0.001, _b_, *p*<0.05). Differences were analyzed using One-way ANOVA and Tukey's post test. (**C**) TNFα secretion from trout macrophages stimulated with liposomes for 16 h was assessed by Western blot. NL_c_ Dose 2 = 375 µg/ml liposomes, 12.5 µg/ml poly (I:C), 6.25 µg/ml LPS, NL_2,n_ = empty liposomes (375 µg/ml) and LPS = stimulation control (6.25 µg/ml). A representative Western Blot is shown.

We examined the gene expression patterns in response to NL_c_ treatment in ZFL cells and trout macrophages (**Figure 5A and 5B**) by evaluating the expression of marker genes of pro-inflammatory (TNFα and IL-6) and anti-viral responses (IFNΦ and α, GIG2 and CCL4). Figure 5A shows that IFNΦ and GIG2 gene expression was significantly induced by the NL_c_ formulation at both doses, but we did not observe significant differences between Dose 1 and 2. Importantly, IFNΦ (NL_c_ Dose 1: 11±2 -fold change; p<0.01) and GIG2 (NL_c_ Dose 1: 2250±49 -fold change; p<0.01) had higher expression levels in NL_c_ formulation than in non-loaded liposomes (NL_2,n_: 5±4 -fold change and 17±1.5 -fold change, respectively). The chemokine CCL4, a chemotactic cytokine that is induced in fish after viral infection [35], was also efficiently induced after NL_c_ treatment (**Figure 5A**). We also observed that non-loaded liposomes (NL_2,n_) were still able to induce low levels of gene expression (**Figure 5A and 5B**). Several groups have indeed described that cationic liposomes have an immunological adjuvant effect and that they are able to regulate the transcription of several chemokines and cytokines [36].

We also assessed the IFNα, IL-6 and TNFα expression levels in trout macrophages (**Figure 5B**) to further evaluate the stimulatory ability of NL_c_ formulation. The IFN expression was significantly induced after NL_c_ Dose 1 and 2 treatment (68±5 and 50±10.5 -fold change; p<0.001) as compared to non-loaded liposomes (NL_2,n_; 9.2±3.8 -fold change; p<0.001) and to the free LPS/poly (I:C) mixture (12±4 -fold change; p<0.001). The pro-inflammatory cytokines IL-6 and TNFα showed a slightly different pattern, achieving good stimulation levels after NL_c_ treatment with respect to non-loaded liposomes, but similar or lower levels when compared to the free-LPS/poly (I:C) mixture (**Figure 5B**). Consistent with gene expression results, TNFα protein secretion was strongly induced by NL_c_ formulation, and most importantly, it was undetectable after stimulation with non-loaded liposomes NL_2,n_ (**Figure 5C**). TNFα is one of the pivotal early response cytokines that are secreted by macrophages and enters the circulation to exert its systemic action [37].

### *In vivo* biocompatibility of the NL_c_ formulation

**Figure 6:**
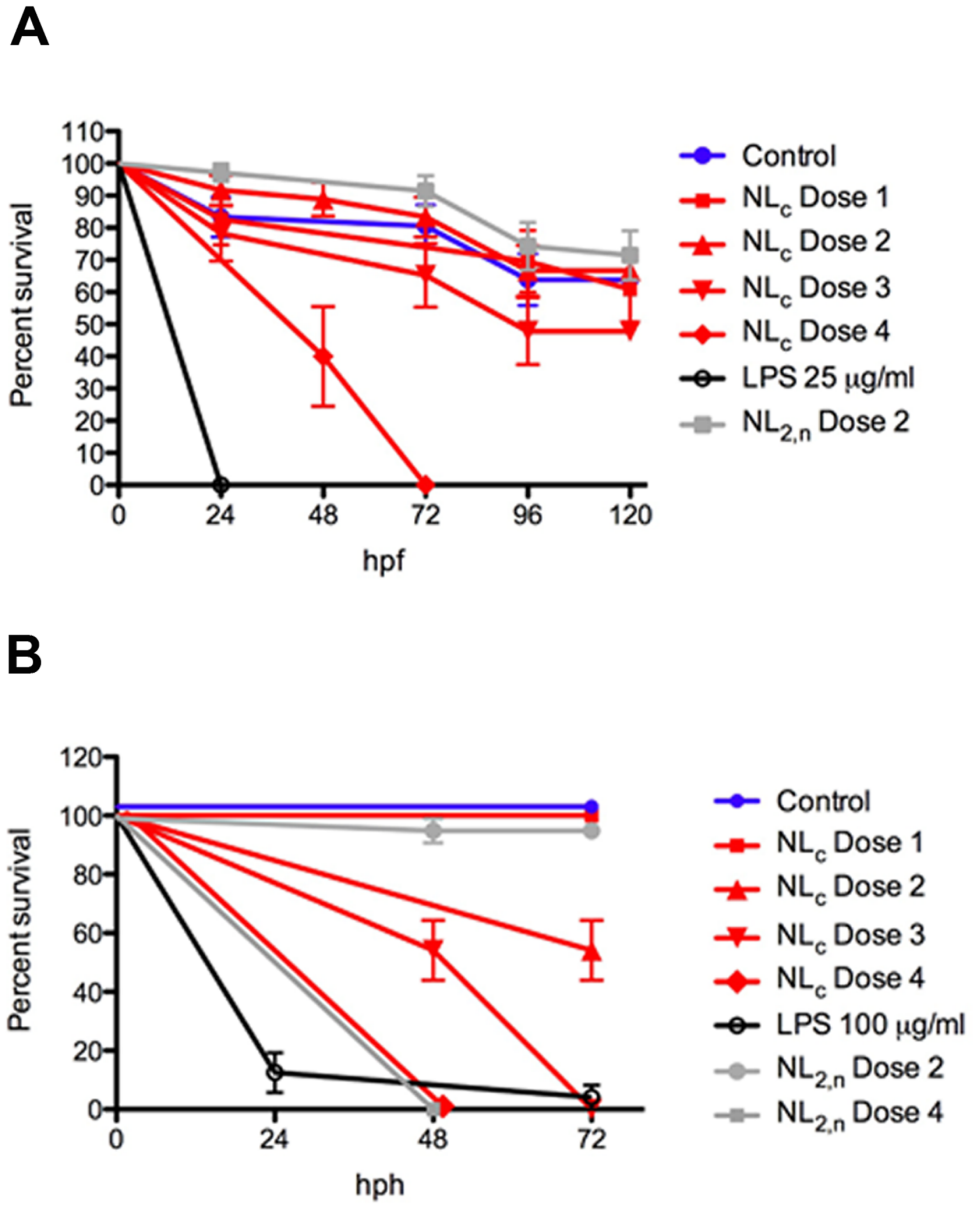
*In vivo* NL_c_ formulation toxicities. Survival of zebrafish embryos was recorded every 24(hpf) (**A**) and 72 h post-hatching (hph) (**B**) after exposure to four concentrations of liposomal IS cocktail (red, NL_c_ Dose 1 = 750 µg/ml liposomes, 25 µg/ml poly (I:C) and 12.5 µg/ml LPS; NL_c_ Dose 2 = 1.5 mg/ml liposomes, 50 µg/ml poly (I:C) and 25 µg/ml LPS; NL_c_ Dose 3 = 3 mg/ml liposomes, 100 µg/ml poly (I:C) and 50 µg/ml LPS; NL_c_ Dose 4 = 6 mg/ml liposomes, 200 µg/ml poly (I:C) and 100 µg/ml LPS). Liposomes without encapsulated immunostimulants (grey, NL_2,n_ Dose 2 = 1.5 mg/ml, NL_2,n_ Dose 4 = 6 mg/ml) and non-treated embryos (blue) were used as controls. Non-encapsulated LPS (black, 25 µg/ml and 100 µg/ml) was used as mortality control. Differences were analyzed using log rank test. *, *p*<0.05; **, *p*<0.01; ***, *p*<0.001.

We conducted different dose-response survival experiments with the NL_c_ formulation and non-loaded liposomes NL_2,n_ in pre- and post-hatching larvae (**Figure 6** **and Figure S6 in File S1**). A NL_c_ concentration range from an extremely high dose (NL_c_ Dose 4 = 6 mg/ml) to a putative therapeutic dose (NL_c_ Dose 1 = 0.75 mg/ml) was chosen. We did not observe significant differences in survival curves obtained with pre-hatched larvae incubated with NL_c_ formulation at different doses (**Figure 6A**), and only very high doses (NL_c_ Dose 4) caused a significant increase in mortality (100% at 72 h, p<0,0001). In contrast, high LPS toxicity with free-LPS treatment both in pre- and post-hatching larvae was observed (**Figure S6A and S6B in File S1**). A moderate poly (I:C) toxicity in pre-hatching larvae (62.5% mortality at 120 hpf; p<0.0001) versus control (36.12% mortality at 120 hpf; p<0.0001) was also recorded. Therefore, and in accordance with our previous *in vitro* toxicity studies (**Figure 2**), the encapsulation of both immunostimulants avoided the embryo/larvae mortality induced by free LPS and poly (I:C) (**Figure 6A** **and Figure S6 in File S1**). Importantly, the embryos incubated with NL_c_ formulations were able to hatch and develop normally until 120 h with no morphological defects. The survival curves in post-hatching larvae incubated with these liposomal formulations were substantially different (**Figure 6B**). In this case, non-loaded liposomes (NL_2,n_ Dose 2, 1.5 mg/ml) showed less toxicity than that of the corresponding liposomal IS-cocktail (NL_c_ Dose 2, 1.5 mg/ml liposomes, 50 µg/ml poly (I:C), 25 µg/ml LPS). In addition, a dose-dependent toxicity for the NL_c_ formulation after 48 h incubation was observed (**Figure 6B**). Analysis of gene expression in NL_c_ challenged larvae at 24, 48 and 72 h showed expression of marker genes of pro-inflammatory (TNFα and iNOS) and anti-viral responses (TLR3 and GIG2) (**Figure S7 in File S1**), indicating a stimulation of the zebrafish larvae immune system. Finally, DLS measurements done using NL_c_ and NL_2,n_ formulations after 5 days incubation in E3 medium indicated a good stability after the *in vivo* challenge. We also characterized the NL_c_ stability in *in vivo* experimental conditions by Turbiscan, and we found that the NL_c_ stability index was not significantly changed after incubation in aquarium water or in E3 medium at 28°C for 2 days (stability indexes of 6.16 and 3.8, respectively). These data further confirm that this liposomal IS-cocktail might be used for future *in vivo* immunization in aquatic species.

## Discussion

Vaccination and preventive immunostimulation has become the principal prophylactic tool for disease control in aquaculture. Some conventional vaccines made with inactivated bacteria (e.g. *Listonella anguillarum* causing vibriosis) have achieved good protection levels against different fish infections [38]. However, most diseases have no prevention tools, causing massive mortalities in fish farms and generating important economic losses. It is still unclear whether teleost fish have immunological memory but the secondary humoral responses are by far less prominent than in mammals [9], [38]. Thus, the activation of the innate immune system seems the most effective way for the initiation of an efficient immune response in fish. The binding of antigens to the innate pathogen receptors (PRRs) located on antigen-presenting cells (APCs) is critical for developing an effective immune response. Fish have a powerful innate immune system with a high molecular diversity and complexity [39], being APCs (especially the macrophages and dendritic cells) the main players of the innate immune response and responsibles for the activation of adaptive immunity [40]. With these specific premises, we have designed a nanosized and non-toxic unilamellar liposomal formulation loaded with TLR ligands (LPS and poly (I:C)) which was able to induce a potent anti-viral and pro-inflammatory response *in vitro* and *in vivo* in fish. As far as we know, this study is the first attempt to co-encapsulate two model immunostimulants specifically designed to target fish APCs in nanosized liposomes. To date, the unique attempt to vaccinate fish using liposomes was done by Irie *et al.*, who explored the use of microsized liposomes containing *A. salmonicida* total extracts in carp [22]. Recently, Fredriksen *et al.* have also shown that a combination of poly(lactic-*co*-glycolic acid) microparticles loaded with β-glucan and human γ-globulins were able to target head kidney macrophages inducing an adaptive *in vivo* immune response in salmon [41]. The LPS would be an excellent candidate for immunostimulation purposes, but it has been scarcely used due to its high endotoxic potential in mammals. Fish are less sensitive to LPS toxic effects [17], and by encapsulating LPS we have assayed a simple way to stimulate fish innate immune system. On the other hand, we also target anti-viral response pathways by adding dsRNA to the nanocarrier [13]. We have achieved high co-encapsulation efficiencies by using liposomes with positive charge that can easily incorporate LPS and poly (I:C) into the lipid bilayer and become neutral liposomes. Although liposomes are in principle highly biocompatible, *in vitro* toxicity of cationic liposomes has been reported by several groups [42], [43]. Thus, the observed charge neutralization has been an advantage, making our formulation highly biocompatible. Another advantage of this encapsulation system has been the elimination of the free LPS associated toxicity observed in cells and larvae (**Figures 2** **and** **6**). The LPS toxicity *in vitro* and *in vivo* has been well documented in different vertebrates [15], and it has also been demonstrated that encapsulation of LPS into liposomes decreased its toxicity compared to the free form [29]. Our system minimizes the detrimental effects of LPS while maintaining the immune system activation potency.

By developing an *in vitro* endocytosis assay with fish cells, we have also demonstrated that NL_c_ liposomes contact with plasma membranes and they are efficiently internalized by fish macrophages and zebrafish hepatocytes. Different studies in rodents and humans have shown the ability of liposomes to deliver different compounds to endosomal compartments [12], [32]. The liposomes developed in this study are 125 nm in size and its endocytosis is inhibited mainly by MβCD and sucrose, which indicates that they likely utilize the caveolae-mediated and the clathrin-mediated endocytosis pathways to reach intracellular compartments. The fact that the NL_c_ liposomes accumulate in endosomal-lysosomal compartments is a potential advantage since TLR3 is located in endosomal membranes, and antigen processing for MHCII presentation takes place in this compartment [3]. In addition, this simple and active formulation designed for virtually all fish species vaccination could be upgraded with specific pathogenic antigens of any particular fish species.

In recent years, health and environmental safety of nanoparticle-based therapeutics is a major concern for nanotechnology that has to be carefully addressed [44]. The zebrafish embryos and larvae have become a reference model for *in vivo* toxicological studies due to its sensitivity and logistic convenience [45]–[47]. Zebrafish embryos are protected from the environment with the chorion, a rigid but permeable membrane, which embryos lose after 48 h (hatching) to become free-swimming larvae [48], [49]. We have taken advantage of the zebrafish model to demonstrate the biocompatibility of our formulation at therapeutic doses and also the ability of NL_c_ to target innate immune system. The activation of the innate immune system in trout macrophages and in zebrafish larvae can be assessed by following the expression of key cytokines [16], [50]. Our study demonstrates that NL_c_ formulation stimulates the expression of several cytokines involved in anti-viral and bacterial response, and in some cases, the treatment with empty NL formulations also stimulates cytokine gene expression. Importantly, TNFα secretion by trout macrophages is potently and specifically stimulated by the liposomal IS-cocktail and not by the non-loaded liposomes. However, several groups have indeed described that cationic liposomes had an immunological adjuvant effect and that they were able to regulate the transcription of different chemokines and cytokines [36]. The induction of specific immune responses with liposomal immunostimulant formulations should be a promising strategy to improve disease control in fish farms.

## Supporting Information

File S1 **Supporting information Table and Figures S1–S7.** **Table S1**. Rainbow trout (*Oncorhynchus mykiss*) and zebrafish (*Danio rerio*) specific primers for PCR and Q-PCR. **Figure S1**. Evaluation of toxicity of cationic liposomes without encapsulated immunostimulants (NL_1,n_ and NL_2,n_). Viability of ZFL cell line was assessed with the MTT assay (**A**) or LDH assay (**B**) after a dose response (0.1 µg/ml-10 mg/ml) with the two liposomal formulations (NL_1,n_ and NL_2,n_). Viability of HepG2 cell line was determined with the MTT assay (**C**) and with the LDH assay (**D**) after a dose response (0.1 µg/ml-10 mg/ml) with the two liposomal formulations (NL_1,n_ and NL_2,n_). Non-treated cells were used as 100% viability control (dotted line) in the MTT assays and non-treated cells were used as control of the basal death (dotted line) in the LDH assays. Data represent means ± SD of three independent experiments. Differences were analyzed using One-way ANOVA followed by Tukey's post test. *, *p*<0.05; **, *p*<0.01; ***, *p*<0.001. **Figure S2**. Cytotoxicity of NL_c_ formulation in ZFL cells by LDH assay. (**A**) Viability of ZFL after 24 h incubation with the liposome-encapsulated LPS (NL_2, LPS_, green bars) at Dose 1 = 1 mg/ml liposome with 50 µg/ml LPS, Dose 2 = 0.5 mg/ml liposome with 25 µg/ml LPS and Dose 3 = 0.20 mg/ml liposome with 10 µg/ml LPS. The white bar is the control treatment with liposomes without encapsulated immunostimulants (NL_2,n_, 1 mg/ml liposome) and the blue bar is the non-encapsulated LPS control (50 µg/ml). (**B**) Viability of ZFL after 24 h incubation with the liposome-encapsulated poly (I:C) (NL_2, poly (I:C)_, green bars) at Dose 1 = 1.5 mg/ml liposome with 50 µg/ml poly (I:C), Dose 2 = 0.75 mg/ml liposome with 25 µg/ml poly (I:C) and Dose 3 = 0.375 mg/ml liposome with 10 µg/ml poly (I:C). The white bar is the control treatment with empty liposomes (NL_2,n_, 1.5 mg/ml liposome) and the red bar is the non-encapsulated poly (I:C) control (50 µg/ml). (**C**) Viability of ZFL cells after 24 h with liposomal LPS-poly (I:C) cocktail (NL_c_, green bars) at Dose 1 = 1.5 mg/ml liposome with 50 µg/ml poly (I:C) and 25 µg/ml LPS, Dose 2 = 0.75 mg/ml liposome with 25 µg/ml poly (I:C) and 12.5 µg/ml LPS and Dose 3 = 0.375 mg/ml liposome with 12.5 µg/ml poly (I:C) and 6.25 µg/ml LPS. The white bar is the control treatment with empty liposomes (NL_2,n_, 1.5 mg/ml liposome), the blue bar is the non-encapsulated LPS (25 µg/ml) and the red bar represents the non-encapsulated poly (I:C) control (50 µg/ml). Non-treated cells were used as 100% viability control (dotted line). Data represent means ± SD of three independent experiments. Differences were analyzed using One-way ANOVA followed by Tukey's post test. *, *p*<0.05; ***, *p*<0.001. **Figure S3**. *In vitro* cytotoxicity of NL_c_ formulation in trout macrophages. (**A**) The cytotoxicity of NL_c_ was assessed by the LDH assay. Viability of the trout macrophage primary cell culture after 24 h incubation with NL_c_ encapsulating both poly (I:C) and LPS (green bars) at Dose 1 = 0.75 mg/ml liposome with 25 µg/ml poly (I:C) and 12.5 µg/ml LPS and Dose 2 = 0.375 mg/ml liposome with 12.5 µg/ml poly (I:C) and 6.25 µg/ml LPS. The white bar is the control treatment with non-encapsulating liposomes (NL_2,n_, 0.75 mg/ml liposome) and the grey bar is the non-encapsulated poly (I:C) and LPS control (25 µg/ml and 12.5 µg/ml, respectively). Basal dead cells of the non-treated cells were used as control (dotted line). Data represent means ± SD of 3 independent experiments. Differences were analyzed using One-way ANOVA followed by Tukey's post test **, *p*<0.01. **Figure S4**. *In vitro* cytotoxicity of endocytosis inhibitors. (**A**) Viability of ZFL cells after 1 h exposure (16 h in the case of the chloroquine) to different endocytosis inhibitors, assessed by the MTT assay. (**B**) Viability of trout macrophages after 1 h exposure to different endocytosis inhibitors, assessed by the MTT assay. Non-treated cells were used as a 100% viability control (Control bar). **Figure S5**. Time-course of NL_c_ uptake *in vitro*. (**A**) Flow cytometry time course of NL_c_ uptake (grey bars, liposomes at 750 µg/ml containing 25 µg/ml poly (I:C) and 12.5 µg/ml LPS) by ZFL cells. To study the metabolization of NL_c_, ZFL cells were also pretreated for 1 h with chloroquine at 100 µM (red bars). Then, liposomes were added (750 µg/ml liposome containing 25 µg/ml poly (I:C) and 12.5 µg/ml LPS), and left to incubate in the constant presence of chloroquine. (**B**) Flow cytometry time course of NL_c_ uptake (grey bars, liposomes at 750 µg/ml containing 25 µg/ml poly (I:C) and 12.5 µg/ml LPS) by trout macrophages. Cells not exposed to NL_c_ were used as controls (white bars). Data represent means ± SD of triplicates of three independent experiments. **Figure S6**. *In vivo* NL_c_ toxicity assay controls. Survival of zebrafish embryos was recorded every 24 h at 120 h post fertilization (hpf) (**A**) and 72 h post hatching (hph) (**B**) after exposure to non-encapsulated LPS (black, 25 µg/ml and 100 µg/ml), non-encapsulated poly (I:C) (green, 50 µg/ml) and non-encapsulated LPS (25 µg/ml) and poly (I:C) (50 µg/ml) in combination (orange). Non-treated embryos (blue) were used as controls. Survival curves were analyzed using the log rank test (n = 24 individual). **Figure S7**. Analysis of gene expression in zebrafish larvae after time-course exposure to liposome preparation. NL_2,n_ = liposomes without encapsulated immunostimulants (1.5 mg/ml), NL_c_ = liposomes (1.5 mg/ml) with 50 µg/ml poly (I:C) and 25 µg/ml LPS and LPS+poly (I:C) = stimulation control (50 µg/ml poly (I:C), 25 µg/ml LPS). Non-treated embryos were used as control (Ctrl). Elongation factor (EF1) was the reference gene and TLR3, GIG2, TNFα and iNOS mRNA abundance was analyzed by conventional PCR (right panel). Representative images of three independent experiments are shown.
(DOCX)
