# Supplementary material for: A Novel Liposome-Based Nanocarrier Loaded with an LPS-dsRNA Cocktail for Fish Innate Immune System Stimulation
Source: PLoS One. 2013 Oct 18;8(10):e76338. doi: 10.1371/journal.pone.0076338 (PMC3799751; doi:10.1371/journal.pone.0076338)
Supplement: File S1 — Supporting information Table and Figures S1–S7. Table S1. Rainbow trout (Oncorhynchus mykiss) and zebrafish (Danio rerio) specific primers for PCR and Q-PCR. Figure S1. Evaluation of toxicity of cationic liposomes without encapsulated immunostimulants (NL1,n and NL2,n). Viability of ZFL cell line was assessed with the MTT assay (A) or LDH assay (B) after a dose response (0.1 µg/ml-10 mg/ml) with the two liposomal formulations (NL1,n and NL2,n). Viability of HepG2 cell line was determined with the MTT assay (C) and with the LDH assay (D) after a dose response (0.1 µg/ml-10 mg/ml) with the two liposomal formulations (NL1,n and NL2,n). Non-treated cells were used as 100% viability control (dotted line) in the MTT assays and non-treated cells were used as control of the basal death (dotted line) in the LDH assays. Data represent means ± SD of three independent experiments. Differences were analyzed using One-way ANOVA followed by Tukey's post test. *, p<0.05; **, p<0.01; ***, p<0.001. Figure S2. Cytotoxicity of NLc formulation in ZFL cells by LDH assay. (A) Viability of ZFL after 24 h incubation with the liposome-encapsulated LPS (NL2, LPS, green bars) at Dose 1 = 1 mg/ml liposome with 50 µg/ml LPS, Dose 2 = 0.5 mg/ml liposome with 25 µg/ml LPS and Dose 3 = 0.20 mg/ml liposome with 10 µg/ml LPS. The white bar is the control treatment with liposomes without encapsulated immunostimulants (NL2,n, 1 mg/ml liposome) and the blue bar is the non-encapsulated LPS control (50 µg/ml). (B) Viability of ZFL after 24 h incubation with the liposome-encapsulated poly (I:C) (NL2, poly (I:C), green bars) at Dose 1 = 1.5 mg/ml liposome with 50 µg/ml poly (I:C), Dose 2 = 0.75 mg/ml liposome with 25 µg/ml poly (I:C) and Dose 3 = 0.375 mg/ml liposome with 10 µg/ml poly (I:C). The white bar is the control treatment with empty liposomes (NL2,n, 1.5 mg/ml liposome) and the red bar is the non-encapsulated poly (I:C) control (50 µg/ml). (C) Viability of ZFL cells after 24 h with liposomal LPS-po [file pone.0076338.s001.docx]

**Supporting information File S1.**

**Table S1**


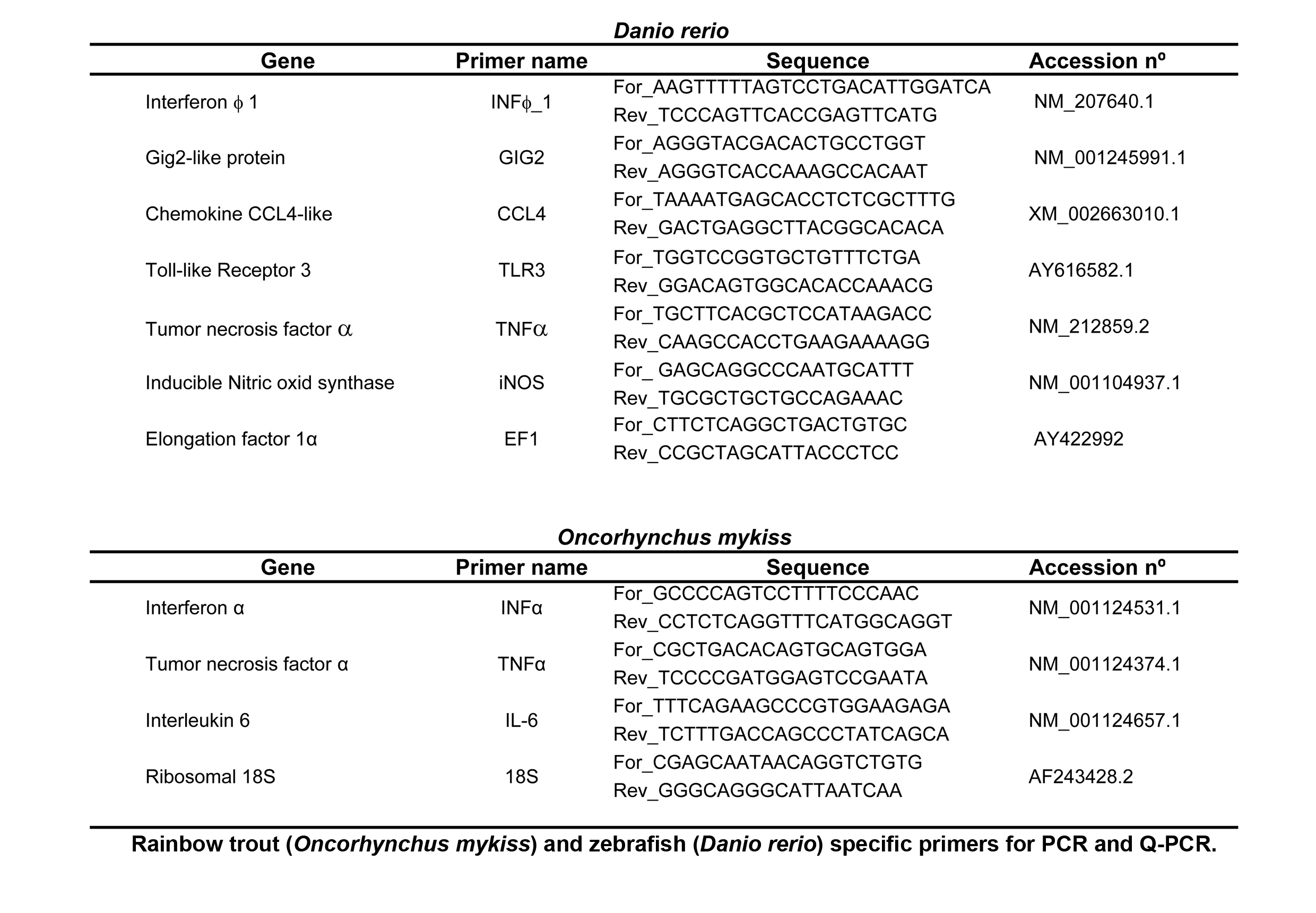


**Figure S1**


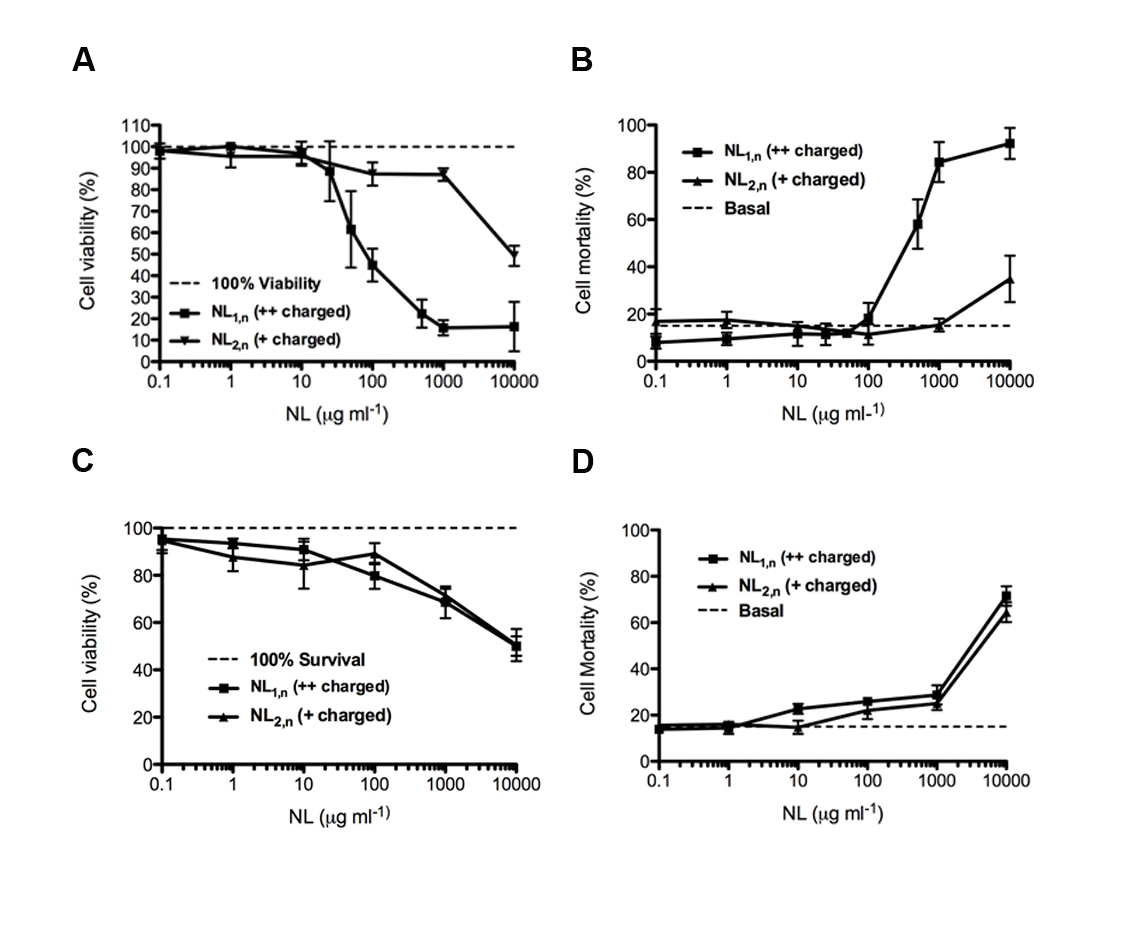


**Figure S2**


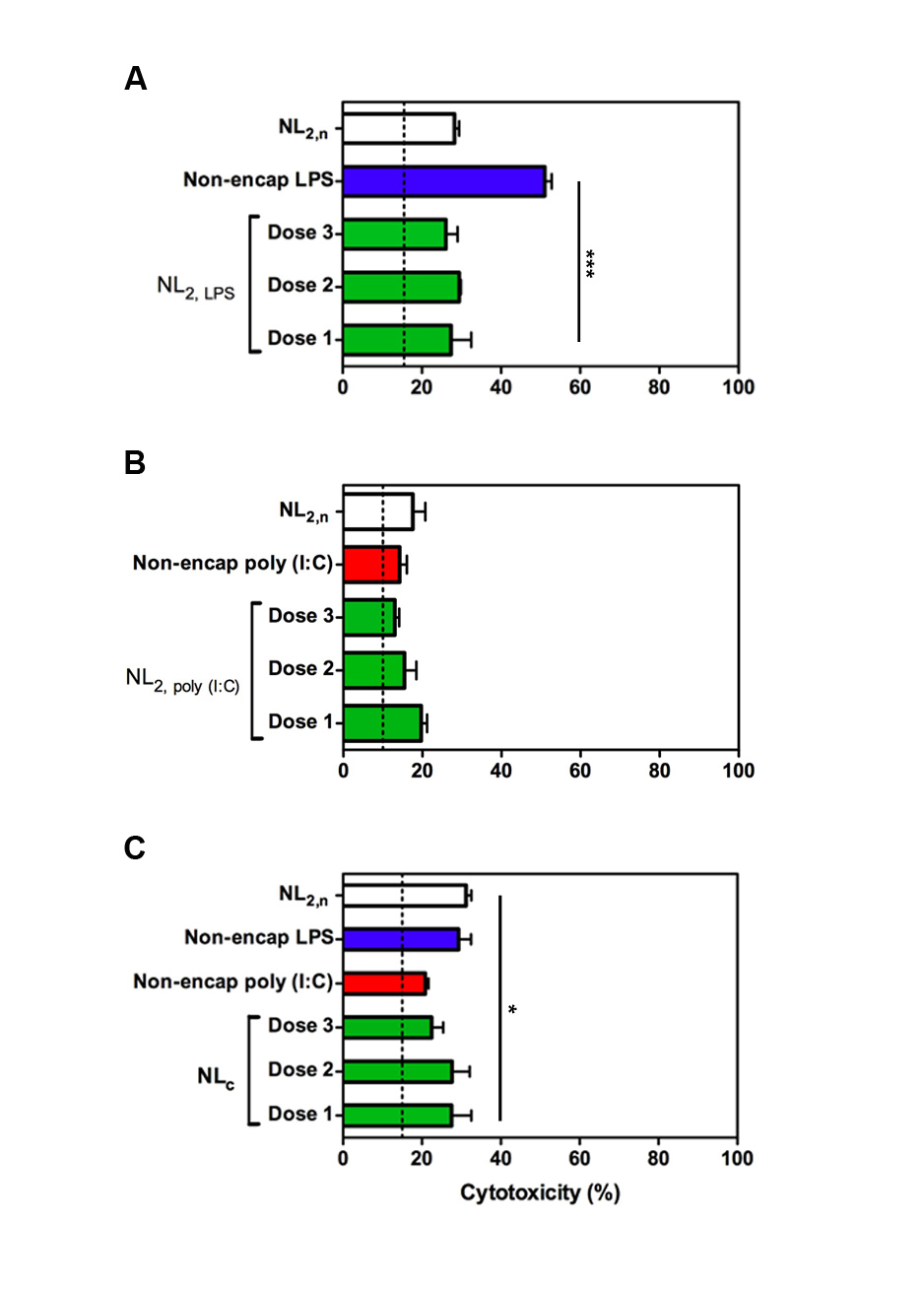


**Figure S3**


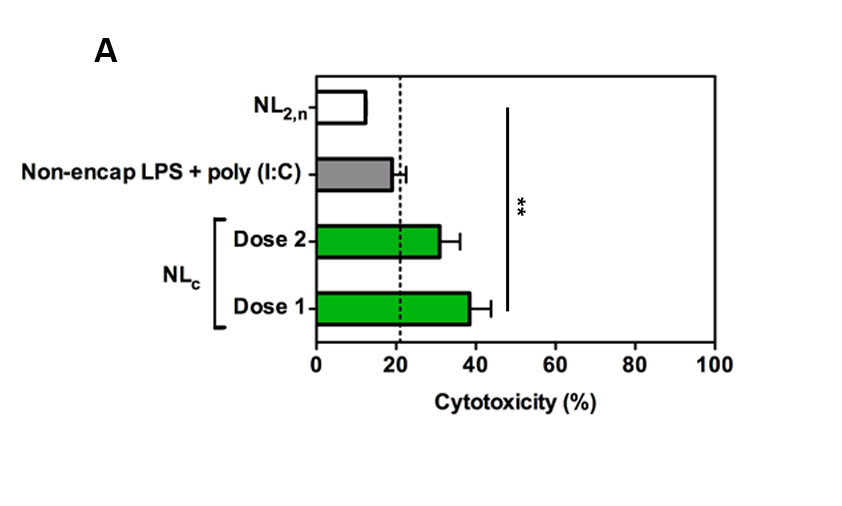


**Figure S4**


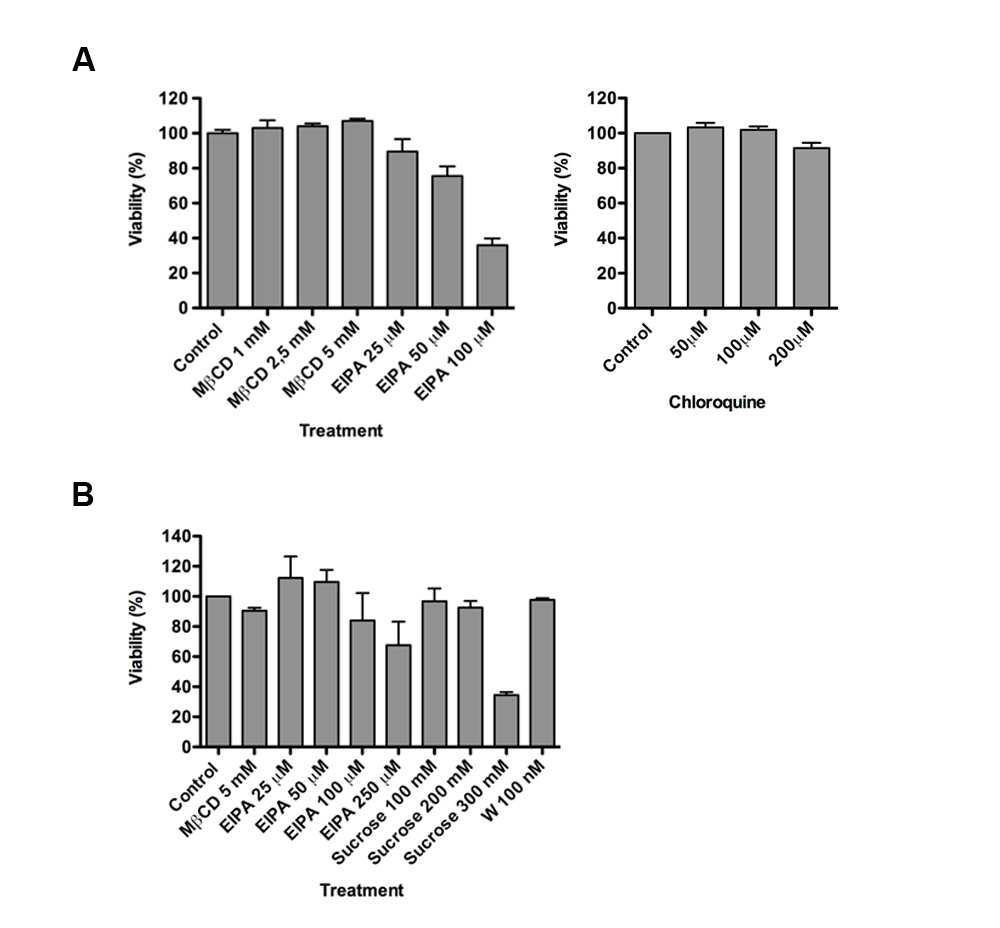


**Figure S5**


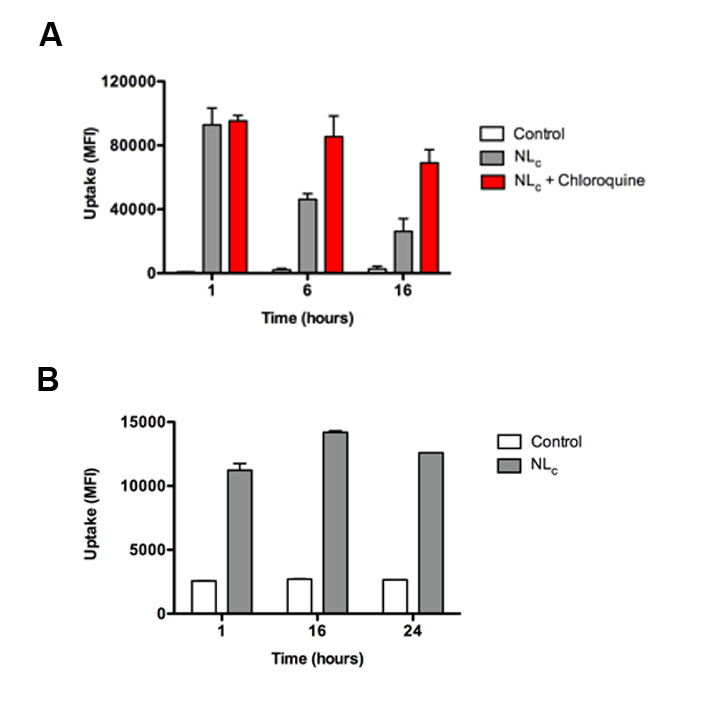


**Figure S6**


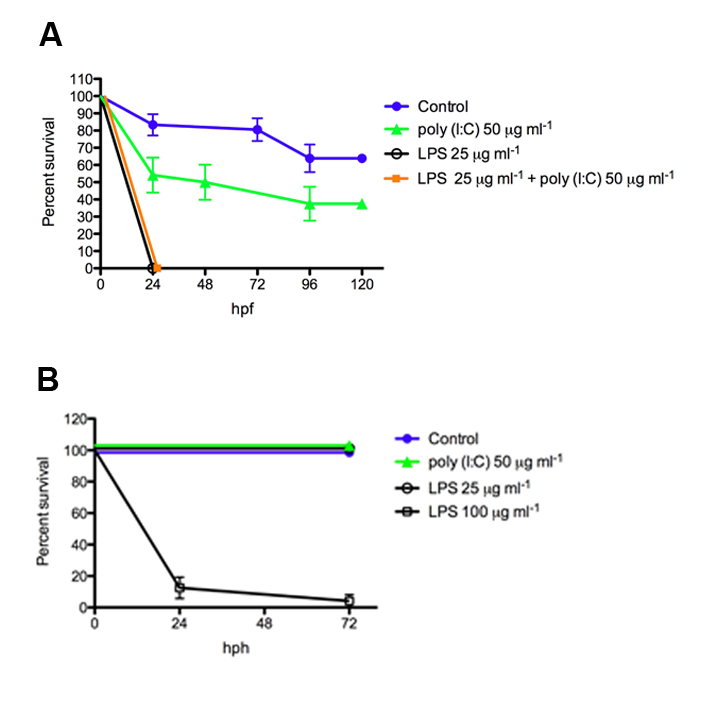


**Figure S7**


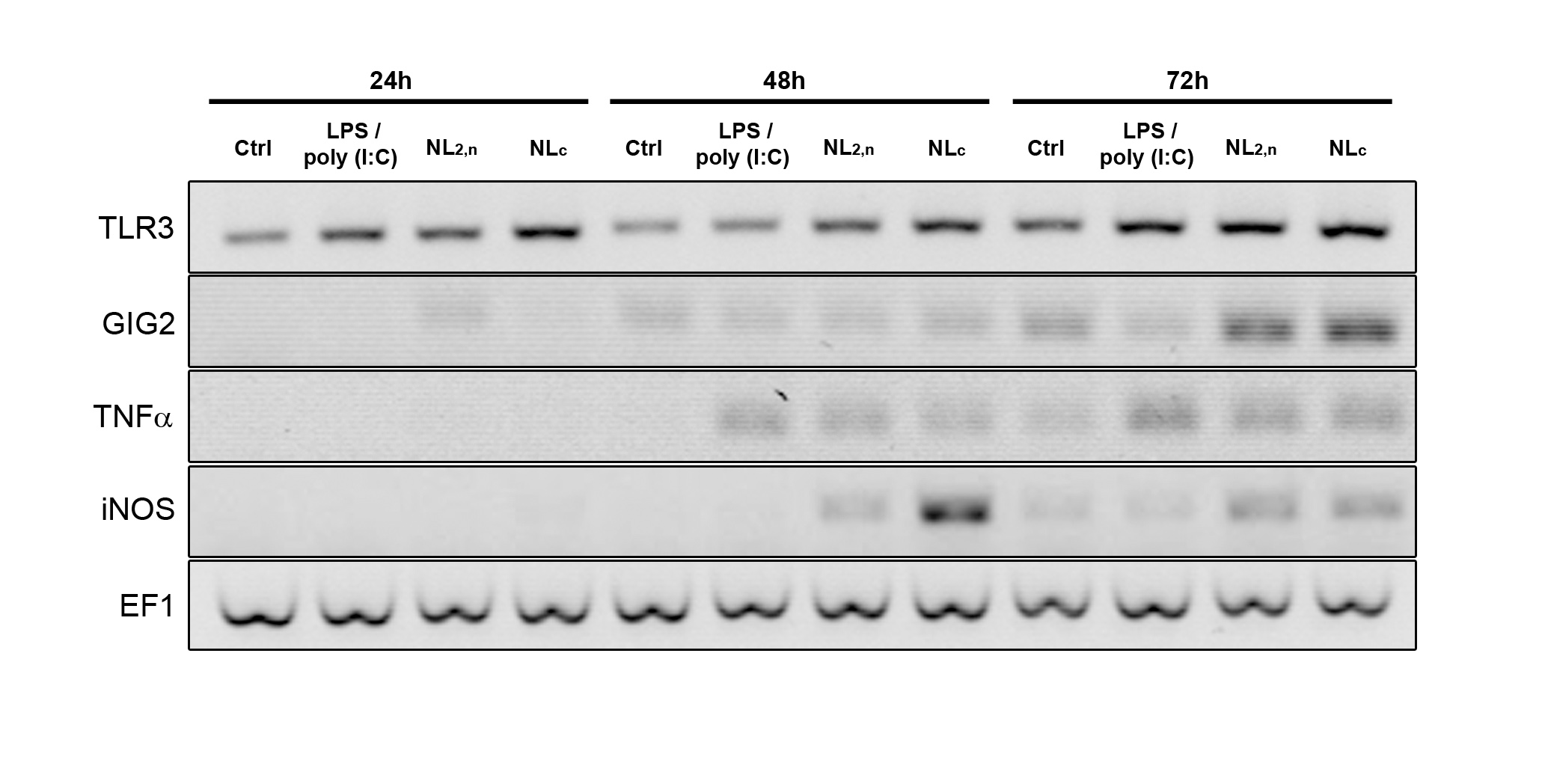


**Supporting Information File S1 Legend**

**Table S1.** Rainbow trout (*Oncorhynchus mykiss*) and zebrafish (*Danio rerio*) specific primers for PCR and Q-PCR.

**Figure S1.** **Evaluation of toxicity of cationic liposomes without encapsulated immunostimulants (NL_1,n_ and NL_2,n_).** Viability of ZFL cell line was assessed with the MTT assay **(A)** or LDH assay **(B)** after a dose response (0.1 µg/ml-10 mg/ml) with the two liposomal formulations (NL_1,n_ and NL_2,n_). Viability of HepG2 cell line was determined with the MTT assay **(C)** and with the LDH assay **(D)** after a dose response (0.1 µg/ml-10 mg/ml) with the two liposomal formulations (NL_1,n_ and NL_2,n_). Non-treated cells were used as 100% viability control (dotted line) in the MTT assays and non-treated cells were used as control of the basal death (dotted line) in the LDH assays. Data represent means ± SD of three independent experiments. Differences were analyzed using One-way ANOVA followed by Tukey’s post test. *, *p <* 0.05; **, *p* *<* 0.01; ***, *p <* 0.001.

**Figure S2.** **Cytotoxicity of NL_c_ formulation in ZFL cells by LDH assay.** **(A)** Viability of ZFL after 24 h incubation with the liposome-encapsulated LPS (NL_2, LPS_, green bars) at Dose 1 = 1 mg/ml liposome with 50 µg/ml LPS, Dose 2 = 0.5 mg/ml liposome with 25 µg/ml LPS and Dose 3 = 0.20 mg/ml liposome with 10 µg/ml LPS. The white bar is the control treatment with liposomes without encapsulated immunostimulants (NL_2,n_, 1 mg/ml liposome) and the blue bar is the non-encapsulated LPS control (50 µg/ml). **(B)** Viability of ZFL after 24 h incubation with the liposome-encapsulated poly (I:C) (NL_2, poly (I:C)_ ,green bars) at Dose 1 = 1.5 mg/ml liposome with 50 µg/ml poly (I:C), Dose 2 = 0.75 mg/ml liposome with 25 µg/ml poly (I:C) and Dose 3 = 0.375 mg/ml liposome with 10 µg/ml poly (I:C). The white bar is the control treatment with empty liposomes (NL_2,n_, 1.5 mg/ml liposome) and the red bar is the non-encapsulated poly (I:C) control (50 µg/ml). **(C)** Viability of ZFL cells after 24 h with liposomal LPS-poly (I:C) cocktail (NL_c_, green bars) at Dose 1 = 1.5 mg/ml liposome with 50 µg/ml poly (I:C) and 25 µg/ml LPS, Dose 2 = 0.75 mg/ml liposome with 25 µg/ml poly (I:C) and 12.5 µg/ml LPS and Dose 3 = 0.375 mg/ml liposome with 12.5 µg/ml poly (I:C) and 6.25 µg/ml LPS. The white bar is the control treatment with empty liposomes (NL_2,n_, 1.5 mg/ml liposome), the blue bar is the non-encapsulated LPS (25 µg/ml) and the red bar represents the non-encapsulated poly (I:C) control (50 µg/ml). Non-treated cells were used as 100% viability control (dotted line). Data represent means ± SD of three independent experiments. Differences were analyzed using One-way ANOVA followed by Tukey’s post test. *, *p <* 0.05; ***, *p <* 0.001.

**Figure S3.** ***In vitro* cytotoxicity of NL_c_ formulation in trout macrophages.** **(A)** The cytotoxicity of NL_c_ was assessed by the LDH assay. Viability of the trout macrophage primary cell culture after 24 h incubation with NL_c_ encapsulating both poly (I:C) and LPS (green bars) at Dose 1 = 0.75 mg/ml liposome with 25 µg/ml poly (I:C) and 12.5 µg/ml LPS and Dose 2 = 0.375 mg/ml liposome with 12.5 µg/ml poly (I:C) and 6.25 µg/ml LPS. The white bar is the control treatment with non-encapsulating liposomes (NL_2,n_, 0.75 mg/ml liposome) and the grey bar is the non-encapsulated poly (I:C) and LPS control (25 µg/ml and 12.5 µg/ml, respectively). Basal dead cells of the non-treated cells were used as control (dotted line). Data represent means ± SD of 3 independent experiments. Differences were analyzed using One-way ANOVA followed by Tukey’s post test **, *p <* 0.01.

**Figure S4. *In vitro* cytotoxicity of endocytosis inhibitors.** **(A)** Viability of ZFL cells after 1 h exposure (16 h in the case of the chloroquine) to different endocytosis inhibitors, assessed by the MTT assay. **(B)** Viability of trout macrophages after 1 h exposure to different endocytosis inhibitors, assessed by the MTT assay. Non-treated cells were used as a 100% viability control (Control bar).

**Figure S5.** **Time-course of NL_c_ uptake *in vitro*.** **(A)** Flow cytometry time course of NL_c_ uptake (grey bars, liposomes at 750 µg/ml containing 25 µg/ml poly (I:C) and 12.5 µg/ml LPS) by ZFL cells. To study the metabolization of NL_c_, ZFL cells were also pretreated for 1 h with chloroquine at 100 µM (red bars). Then, liposomes were added (750 µg/ml liposome containing 25 µg/ml poly (I:C) and 12.5 µg/ml LPS), and left to incubate in the constant presence of chloroquine. **(B)** Flow cytometry time course of NL_c_ uptake (grey bars, liposomes at 750 µg/ml containing 25 µg/ml poly (I:C) and 12.5 µg/ml LPS) by trout macrophages. Cells not exposed to NL_c_ were used as controls (white bars). Data represent means ± SD of triplicates of three independent experiments.

**Figure S6. *In vivo* NL_c_ toxicity assay controls**. Survival of zebrafish embryos was recorded every 24 h at 120 h post fertilization (hpf) **(A)** and 72 h post hatching (hph) **(B)** after exposure to non-encapsulated LPS (black, 25 µg/ml and 100 µg/ml), non-encapsulated poly (I:C) (green, 50 µg/ml) and non-encapsulated LPS (25 µg/ml) and poly (I:C) (50 µg/ml) in combination (orange). Non-treated embryos (blue) were used as controls. Survival curves were analyzed using the log rank test (n=24 individual).

**Figure S7. Analysis of gene expression in zebrafish larvae after time-course exposure to liposome preparation.** NL_2,n_ = liposomes without encapsulated immunostimulants (1.5 mg/ml), NL_c_ = liposomes (1.5 mg/ml) with 50 µg/ml poly (I:C) and 25 µg/ml LPS and LPS + poly (I:C) = stimulation control (50 µg/ml poly (I:C), 25 µg/ml LPS). Non-treated embryos were used as control (Ctrl). Elongation factor (EF1) was the reference gene and TLR3, GIG2, TNFα and iNOS mRNA abundance was analyzed by conventional PCR (right panel). Representative images of three independent experiments are shown.
